# Unilateral Acute Serous Retinal Detachment with Pachychoroid Following Postpartum Haemorrhage: A Case Report

**DOI:** 10.1055/a-2542-5369

**Published:** 2025-04-16

**Authors:** Enrico Meduri, Ariane Malclès, Gabriele Thumann, Giorgio Enrico Bravetti

**Affiliations:** 1 Ophthalmology, Hopitaux Universitaires Geneve, Switzerland; 2 Ophthalmology, University of Geneva, Faculty of Medicine, Switzerland

**Keywords:** retina, pachychoroid, pregnancy, subretinal fluid, retinal vascular disease, OCT, Retina, Pachychoroid, Schwangerschaft, subretinale Flüssigkeit, retinale Gefäßerkrankung, OCT

## Abstract

**Purpose** To elucidate the development and resolution of a unilateral acute serous retinal detachment (SRD) in a healthy patient following a complicated postpartum haemorrhage. This underscores the impact of systemic volume alterations and stress-induced factors on retinal fluid balance.

**Background** Postpartum SRD is observed in individuals with pre-eclampsia and patients with previously diagnosed central serous chorioretinopathy, both attributed to hormonal and volumetric fluctuations during pregnancy.

**Case Description** A 33-year-old woman presented with metamorphopsia and blurry vision in her left eye 24 hours following childbirth complicated by haemorrhagic shock. Notably, the patient had pre-delivery physiological vital signs with no alterations of consciousness, arterial blood pressure, or renal function. At presentation, the visual acuity in the left eye was 1.0 decimals and the anterior segment was within normal limits, while fundoscopy revealed a slight alteration in the foveal reflex. Spectral-domain optical coherence tomography (SD-OCT) revealed the presence of a dome-shaped SRD in the foveal region with a central foveal thickness of 279 µm and pachychoroid at 410 µm retro-foveally. The examination of the right eye and SD-OCT were unremarkable, except for the presence of pachychoroid at 367 µm retro-foveally. No treatment was initiated. At 48 hours, SD-OCT scans revealed complete resorption of the SRD in the left eye with a persistent focal alteration in the foveal region of the ellipsoid zone. Retro-foveal choroid thickness (RCT) was unchanged. At one month, visual acuity remained stable, and the patient was no longer symptomatic. The left eye SD-OCT revealed a reduction in CFT (279 µm vs. 224 µm, a 20.2% reduction) and RCT (410 µm vs. 360 µm, a 14.6% reduction) compared to baseline. Remarkably, the right eye also exhibited a 14.9% reduction in RCT (367 µm vs. 309 µm).

**Conclusion** This case highlights the role that postpartum systemic changes and complications can play in the occurrence of retinal and choroidal changes. We believe that in this specific case, the development of acute SRD was probably due to oncotic fluctuations related to the hypovolaemic status following postpartum haemorrhage. This also emphasises the utility of SD-OCT for assessment and follow-up monitoring, providing valuable insights into retinal and choroidal changes over time.

## Zusammenfassung

**Fallbeschreibung** Dieser Fallbericht beschreibt eine einseitige akute seröse Netzhautablösung (SRD) mit Pachychoroid bei einer 33-jährigen, gesunden Patientin nach postpartaler Hämorrhagie. Die natürliche Entbindung verlief komplikationsbehaftet, da eine schwere Blutung (ca. 3 l) infolge von Uterusatonie auftrat, was den Einsatz von 4 Bluttransfusionen und 2 Dosen des PGE2-Analogons Sulprostone (0,5 mg) erforderte. Innerhalb von 24 Stunden nach der Geburt klagte die Patientin über Metamorphopsie und verschwommenes Sehen im linken Auge, während die Sehschärfe (1,0 Dezimal) und der vordere Augenabschnitt unauffällig blieben. Die Funduskopie des linken Auges zeigte nur subtile Veränderungen des Fovealreflexes. Die Spektral-Domänen-OCT (SD-OCT) mit erweiterter Tiefenbildgebung (EDI) ergab im linken Auge einen kuppelförmigen SRD im Foveabereich mit einer zentralen Fovealdicke von 279 µm und einer retrofovealen Choroidal-Dicke von 410 µm. Das rechte Auge wies einen Pachychoroid (367 µm) ohne SRD auf. Aufgrund des milden Symptomprofils wurde zunächst keine Therapie eingeleitet, und die Patientin wurde überwacht. Bereits 48 Stunden später zeigte eine Wiederholungsuntersuchung eine vollständige Resorption des SRD, wenngleich eine fokale Störung der Ellipsoidzone fortbestand. Beim einmonatigen Follow-up reduzierten sich sowohl die zentrale Fovealdicke als auch die Choroidal-Dicke signifikant, und die Ellipsoidzone stellte sich vollständig wieder her. Sechs Monate nach der Erstvorstellung sank die retrofoveale Choroidal-Dicke beidseits weiter.

**Schlussfolgerung** Der Bericht legt nahe, dass die SRD durch onkotische Schwankungen infolge der hypovolämischen Situation nach postpartaler Hämorrhagie ausgelöst wurde – möglicherweise begünstigt durch die verabreichten PGE2-Analoga. Dieser Fall unterstreicht den Einfluss systemischer Flüssigkeitsverschiebungen und hormoneller Veränderungen auf die Netzhautfunktion sowie den diagnostischen Wert der SD-OCT im EDI-Modus.

## Introduction

Postpartum serous retinal detachment (SRD) is a very rare condition. It is typically associated with systemic conditions during pregnancy, such as (pre-)eclampsia, hypertension, HELLP syndrome (haemolysis, elevated liver enzymes, and low platelets), disseminated intravascular coagulation, and thrombotic thrombocytopenic purpura. Additionally, SRD can present as an exacerbation of preexisting retinal pathologies including diabetic retinopathy and central serous chorioretinopathy (CSCR). These are linked to hormonal and fluid volume changes during pregnancy 1 , 2 . The rarity of this condition in healthy postpartum patients emphasises the complexity of underlying mechanisms. In most instances, SRD emerges in the third trimester and resolves spontaneously within a few months postpartum 3 . However, the unpredictable nature of SRD during pregnancy can sometimes pose diagnostic and therapeutic challenges for clinicians, particularly when it overlaps with other systemic conditions that complicate management.

To the best of our knowledge, this report, written with patientʼs informed consent, is the first description of an acute unilateral SRD associated with pachychoroid after post-partum haemorrhage (PPH) in an otherwise healthy patient, resolving spontaneously with full visual recovery.

Furthermore, we aim to explore the potential mechanisms underlying SRD development in such cases as well as the diagnostic and management role of spectral domain optical coherence tomography (SD-OCT) enhanced deep imaging (EDI), which offers crucial insights into retinal and choroidal changes.

## Case Description

A 33-year-old healthy female presented with metamorphopsia and blurry vision in her left eye 24 hours after a natural childbirth complicated by post-partal haemorrhage due to uterine atony. This severe haemorrhage, resulting in approximately 3 litres blood loss, necessitated the transfusion of 4 units of blood and administration of 2 doses of sulprostone 0.5 mg, a prostaglandin E2 analogue (PGE2-analog), to control the haemorrhage. Notably, the patient had an unremarkable medical and ophthalmological history, and her pregnancy course had been normal prior to delivery with no evidence of hypertensive disorders or other complications.

**Fig. 1:**
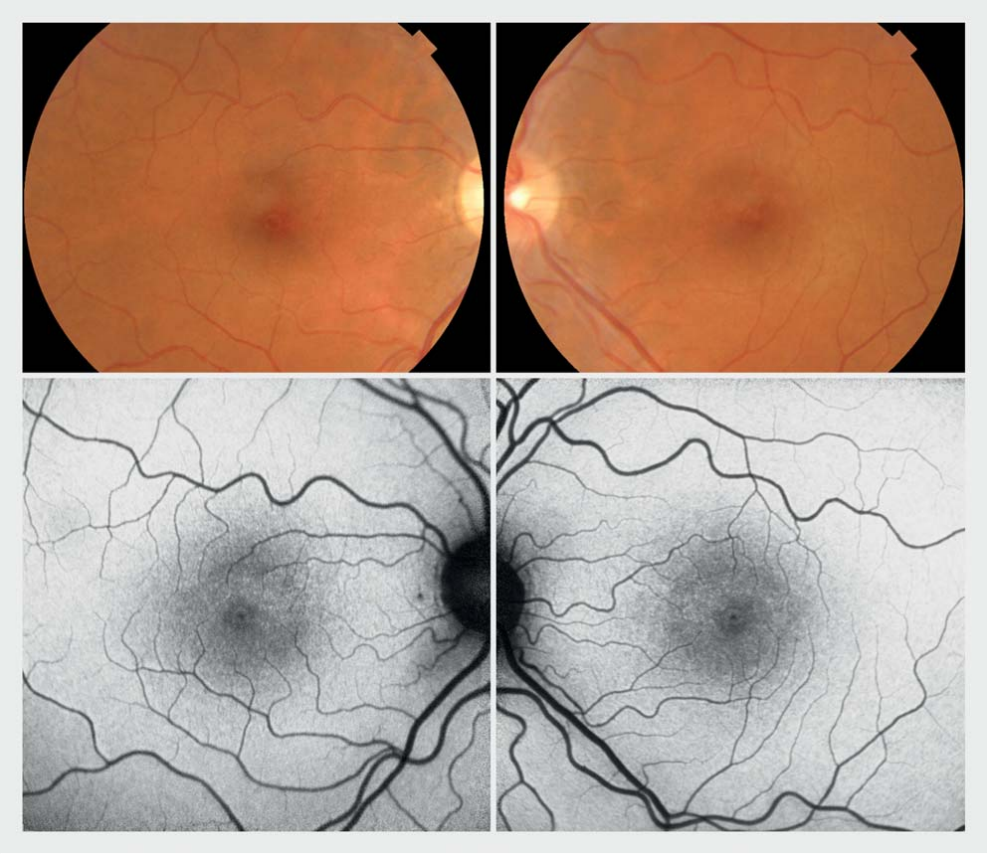
Fundoscopy and autofluorescence photographs of both eyes.

On examination, her visual acuity was 1.0 decimal in both eyes. Anterior segment examination and intraocular pressure were within normal limits bilaterally. Fundoscopy of the left eye revealed subtle alteration of the foveal reflex without any signs of inflammation, while the right eye appeared physiologically normal. Left eye SD-OCT EDI showed a dome-shaped SRD without pigment epithelial detachment, with a central foveal thickness (CFT) of 279 µm and a retro-foveal choroidal thickness (RCT) of 410 µm. SD-OCT EDI scan of the right eye was unremarkable except for the presence of a pachychoroid measuring 367 µm subfoveally. Autofluorescence was normal for both eyes ( Fig. 1 ). Given the preserved visual acuity and mild nature of the patientʼs symptoms, no treatment was initiated, and the patient was closely monitored.

Forty-eight hours after presentation, repeat SD-OCT EDI scan revealed complete resolution of the SRD in the left eye, with residual focal granular alteration in the foveal region and an interruption of the ellipsoid zone. Retro-foveal choroidal thickness increased slightly to 379 µm in the right eye and 433 µm in the left eye. Despite these anatomical improvements, the patientʼs visual acuity remained stable, though she continued to report mild symptoms.

At one-month follow-up, the patient reported no further visual disturbances, visual acuity remained unchanged in both eyes, and there was no recurrence of SRD. The CFT in the left eye decreased by 20.2% to 224 µm, and RCT lessened by 14.6% to 360 µm compared to baseline. Furthermore, the outer-retina modifications at the foveal region, including the disruption of the ellipsoid zone, had significantly improved during the follow-up, showing a complete restoration and integrity of the ellipsoid zone at one month. Interestingly, the right eye also exhibited a notable reduction in RCT, decreasing from 367 µm to 309 µm (a 14.9% reduction).

**Fig. 2:**
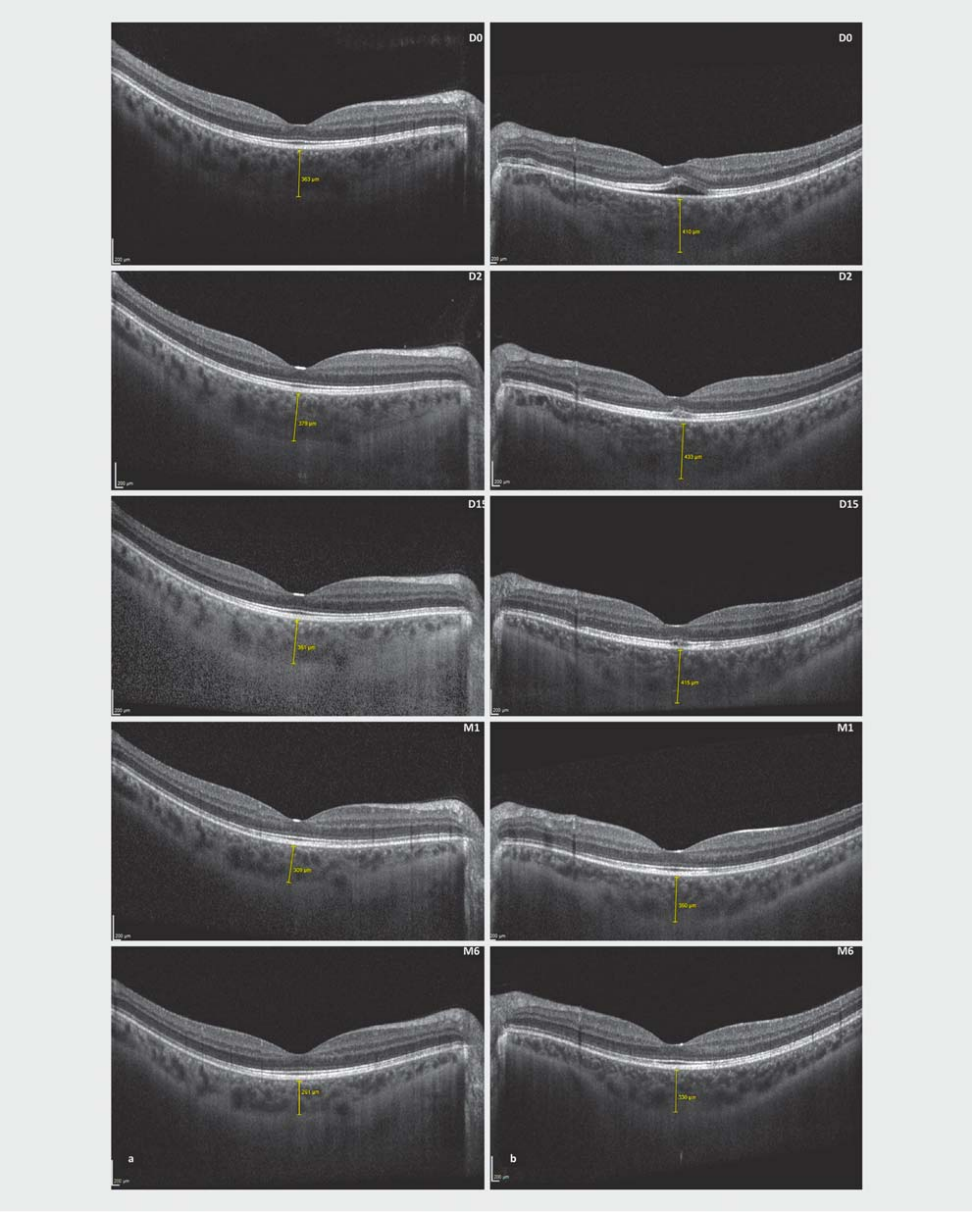
Spectral-domain optical coherence tomography enhanced depth imaging of macular and choroid evolution in both eyes ( **a:** right eye, **b:** left eye). D: days, M: months.

Further follow-up at six months revealed continued bilateral reduction in retro-foveal choroidal thickness with a 28.1% decrease in the right eye and a 19.5% decrease in the left eye compared to baseline ( Fig. 2 ). This gradual decrease in choroidal thickness correlated with the sustained resolution of symptoms and retinal changes.

To ensure consistency, all SD-OCT EDI scans were acquired at similar times of day, between 10 a. m. and 11 a. m., to minimise diurnal variation in choroidal measurements.

## Discussion

This report describes the acute onset of SRD with pachychoroid alteration in a healthy postpartum patient following severe postpartum haemorrhage. Remarkably, the SRD resolved completely within 48 hours in the left eye, accompanied by a comparable total RCT reduction from 410 µm to 360 µm (14.6%) in the left eye and from 367 µm to 309 µm (14.9%) in the right eye at one month. The SRD was associated with outer-retina modifications at the foveal region, including an interruption of the ellipsoid zone, which fully resolved by one-month follow-up.

The choroid, a highly vascularised structure, is essential for maintaining retinal pigment epithelium (RPE) health and outer retina stability. It plays a crucial role in fluid regulation mediated by hydrostatic and osmotic pressure gradients influenced by systemic factors 4 . In cases of acute fluid dysregulation, such as SRD, the balance between these factors can become disrupted, leading to the accumulation of subretinal fluid.

Hypovolaemia secondary to PPH has been shown to redirect blood flow away from peripheral tissues toward critical organs, potentially causing ischaemic damage to visceral and ocular structures 5 , 6 . We hypothesise that this hypovolaemic change impaired choroidal circulation, leading to transient dysfunction of the choroidal drainage system and the RPEʼs pumping mechanism. This disruption likely resulted in fluid stasis, contributing to SRD and pachychoroid formation. Supporting this hypothesis, SD-OCT EDI on the second day after presentation demonstrated an angular sign of Henle fibre layer hyperreflectivity in the inter-papillomacular region of the left eye and an increase in RCT thickness in both eyes at 4.2% in the right eye and 5.3% in the left eye ( Fig. 2 ). This suggests a compensatory response to ischaemic effects on the deep retinal capillary plexus and choriocapillaris (CC), further supporting the idea of hypovolaemia-induced ocular changes 7 .

Additionally, the vasoconstrictive and pro-inflammatory effects of the PGE _2_ analogue sulprostone administered to the patient to manage PPH may have further exacerbated alterations between the CC and RPE fluid dynamics. Prostaglandins have been implicated in exacerbating SRF accumulation, even at lower systemic or topical doses, in conditions such as CSCR 8 , 9 . Specifically, topical latanoprost has been associated with SRF and macular oedema formation. The role of sulprostone in this case aligns with these finding, particularly at the obstetric dose of 1,500 µm administered intravenously.

A synergistic factor may have been the elevated levels of corticosteroids during pregnancy, which can surge following severe stress responses like PPH. Increased endogenous corticosteroids are known to play a crucial role in CSCR development, particularly during the late third trimester. ^9^ These hormonal fluctuations, combined with the osmotic and hydrostatic changes due to hypovolaemia, likely contributed to the transient pachychoroid and SRD 10 .

At 6 months after presentation, the pachychoroid resolved completely, indicating a rebalancing of hormonal, osmotic, and hydrostatic factors without relapses, and a return to normal fluid regulation. Furthermore, we believe that the asymmetry in choroidal thickness between the left and right eyes observed at baseline and over time may have also influenced the unilateral expression of SRD ( Fig. 2 ). This asymmetry in choroidal structure likely predisposed the left eye to SRD development under systemic stress conditions like PPH.

Literature on this topic remains scarce, with only one previous report documenting the onset and resolution of unilateral acute SRD associated specifically with ischaemic choroidopathy without focus on pachychoroid formation in a healthy patient following severe PPH 11 .

In conclusion, this report highlights the potential impact of postpartum systemic changes and complications on occurrence of retinal and choroidal changes. In this specific case, we believe that the development of acute SRD was probably due to oncotic and fluid fluctuations related to the hypovolaemic state following postpartum haemorrhage. Additionally, the administration of PGE2-analogs may have exacerbated retinal fluid dysregulation through mechanisms similar to those observed in CSCR, emphasising the potential impact of prostaglandins in SRF formation, even at lower doses. This report underscores the value of SD-OCT, particularly using enhanced depth imaging mode, in the non-invasive diagnosis and monitoring of macular diseases. SD-OCT offers valuable insights into retinal and choroidal fluid dynamics, making it a critical tool in assessing ocular complications after systemic events such as PPH. Further research is warranted to confirm these anecdotal observations and better understand the clinical relevance of SD-OCT EDI in assessing and managing retinochoroidal changes associated with systemic stress events like PPH. A deeper understanding of these mechanisms may help guide future management strategies for similar cases.
